# The ethics of aggregation in cost-effectiveness analysis or, “on books, bookshelves, and budget impact”

**DOI:** 10.3389/frhs.2022.889423

**Published:** 2022-10-13

**Authors:** Victoria Charlton

**Affiliations:** Department of Global Health and Social Medicine, King's College London, London, United Kingdom

**Keywords:** cost-effectiveness analysis, affordability, budget impact, distributive justice, healthcare priority-setting, ethics, opportunity cost

## Abstract

In deciding how to allocate resources, healthcare priority-setters are increasingly paying attention to an intervention's budget impact alongside its cost-effectiveness. Some argue that approaches that use budget impact as a substantive consideration unfairly disadvantage individuals who belong to large patient groups. Others reject such claims of “numerical discrimination” on the grounds that consideration of the full budget impact of an intervention's adoption is necessary to properly estimate opportunity cost. This paper summarizes this debate and advances a new argument against modifying the cost-effectiveness threshold used for decision-making based on a technology's anticipated budget impact. In making this argument, the paper sets out how the apparent link between budget impact and opportunity cost is largely broken if the effects of a technology's adoption are disaggregated, while highlighting that the theoretical aggregation of effects during cost-effectiveness analysis likely only poorly reflects the operation of the health system in practice. As such, it identifies a need for healthcare priority-setters to be cognizant of the ethical implications associated with aggregating the effects of a technology's adoption for the purpose of decision-making. Throughout the paper, these arguments are illustrated with reference to a “bookshelf” analogy borrowed from previous work.

## Introduction

In setting priorities for spending within a resource-constrained healthcare system, policymakers are generally motivated in part by the goal of efficiency: a distribution of resources that maximizes the good that can be derived from their employment (1–5). Ethically speaking, this is a straightforward if contentious goal that recognizes the moral claim on resources held both by those who stand to benefit from access to new interventions, and those who will suffer as a result of those resources not being put to other uses. Cost-effectiveness analysis is a tool that enables those responsible for decision-making to weigh these claims against one another (6).

If we are to accept that the primary purpose of healthcare is to deliver health, then most healthcare systems are far from achieving perfect efficiency^1^. But policymakers can seek to improve on this situation–normatively speaking–by limiting the adoption of new interventions to those whose use will likely lead to a net increase in population health. Ideally, this calculus would draw on a direct estimate of opportunity cost; that is, the “lost” health associated with the most highly valued alternative use of resources. However, in the absence of such difficult to acquire empirical information, policymakers are frequently guided by the more generic construct of the cost-effectiveness threshold: the theoretical maximum cost per unit health at which opportunity cost is less than or equal to the expected health benefit.

If the cost-effectiveness threshold used for policymaking accurately reflects opportunity cost, and if the health system is proficient in identifying marginally cost-effective activities that can be displaced to fund new interventions, then adopting interventions whose cost per unit health is below the threshold should maintain or improve the system's overall efficiency (13–15). Decisions to adopt such interventions can therefore be clearly justified in ethical terms with reference to the normative goal of efficiency. However, the ethical situation becomes more complex in cases when a decision is made either to adopt an intervention that is not judged to be cost-effective (based on the accepted policy threshold), or not to adopt an intervention that is. This latter situation is relatively rare compared with the former but may occur in cases when an intervention's impact on the healthcare budget is exceptionally large. In such cases, the sheer size of the associated opportunity cost, and the challenge of identifying and divesting from a sufficient number of other health-generating activities in order to free up the necessary resources, has led some health systems to treat such interventions as “unaffordable,” despite their apparent cost-effectiveness (16–21).

The challenge posed by interventions that are cost-effective but unaffordable was brought to the fore in many health systems in the early 2010s by the launch of sofosbuvir. A highly effective, curative treatment for hepatitis C, sofosbuvir was judged by many health systems to be cost-effective, but full adoption was hindered by its huge potential impact on healthcare budgets. Research by the World Health Organization found that the total cost of treating all eligible patients would be equal to at least a tenth of the current annual medicines bill in each of the 30 countries studied, with full provision of the drug threatening health system sustainability in many countries, including relatively wealthy OECD members such as Italy, New Zealand and Japan (22).

In addressing this relatively new type of challenge, countries have adopted a range of approaches. In Europe, strategies aimed at managing the impact of “unaffordable” interventions have included delayed prescription, limits on target patient numbers and revenue caps, as well as collective negotiation with manufacturers in an attempt to reduce prices (23). However, while strategies such as delaying or restricting access to interventions that carry a high opportunity cost may appear pragmatic and, indeed, necessary, it has been argued that they may also be ethically problematic.

In 2016, shortly after its appraisal of sofosbuvir, the UK's National Institute for Health and Care Excellence (NICE), at the request of NHS England, proposed an amendment to its approach that could facilitate delayed adoption of technologies whose potential budget impact exceeds £20 million per year. In response, critics highlighted that such approaches systematically disadvantage members of large patient groups, whose individual treatment costs are aggregated in the calculation of budget impact (15). The authors argued that “the prevalence of someone's condition should not determine their access to treatment” and stated that such policies were open to claims of “numerical discrimination” (16). In a later paper, the same group of authors went on to provide a moral derivation for their opposition to numerical discrimination, arguing that partial provision of “unaffordable” interventions would be preferable to approaches that systematically disadvantage patients based on the morally arbitrary criterion of group size (24). Others, however, have argued that this position fails to recognize the proportionately greater opportunity cost associated with large budget impact, with researchers increasingly devoting attention to methods that allow affordability concerns to be directly incorporated within cost-effective analysis through, amongst other things, modification of the cost-effectiveness threshold (3, 17, 20, 25–27).

This paper summarizes these arguments and advances the position against threshold modification based on anticipated budget impact. In making this argument, the paper sets out how the apparent link between budget impact and opportunity cost is largely broken if the effects of a technology's adoption are disaggregated, while highlighting that the theoretical aggregation of effects during cost-effectiveness analysis is unlikely to be reflected by the operation of the health system in practice. As such, it identifies a need for healthcare priority-setters to be cognizant of the ethical implications associated with aggregating the effects of a technology's adoption for the purpose of decision-making. Throughout the paper, these arguments are illustrated with reference to a “bookshelf” analogy borrowed from previous work (13).

## Opportunity cost and affordability

It is easy to reject the argument that interventions that are “unaffordable” should nonetheless be adopted by resource-limited health systems: surely, if one does not have the resources available to purchase something, one simply cannot purchase it. However, in the context of healthcare priority-setting, the notion of affordability (and its relationship to opportunity cost) is more nuanced than this conclusion would allow. This relationship can be helpfully illustrated through an analogy^2^.

Imagine a large pile of books that you have been tasked with placing on a shelf in such a way as to maximize the total height of the shelved books. Assuming that the shelf is not long enough to accommodate all of the books, an effective way of achieving this outcome would be to rank the books according to height, placing the tallest book on the shelf first and proceeding in rank order until the shelf has been filled. At this point, there is no space for any more books and substituting any shelved book with a replacement from the remaining pile would reduce the total height of the books on the shelf. Going forward, newly purchased books will warrant a place on the shelf only if they are at least as tall as the shortest book, currently positioned at its edge. Otherwise, their addition would lead to an overall reduction in the height of the shelved books.

An analogy's beauty often lies in its obviousness, and the parallels between our hypothetical bookshelf and an efficiency-based approach to healthcare priority-setting should hopefully be reasonably clear. In this analogy, the length of the bookshelf represents the total budget available within a given health system. The range of health interventions available to the system are represented by individual books, with the thickness of each book corresponding to the intervention's budget impact. The height of each book corresponds to the health benefit it offers per unit cost. The aim of maximizing the height of the books on the shelf therefore represents an attempt to maximize efficiency. On a shelf that is already fully laden with books, any additional books that are placed on the shelf carry an opportunity cost: the existing books that must be removed (or the new books that must remain on the pile) to make space for them. Newcomers should therefore only be shelved if they are at least as tall as the shortest book currently placed: a cut-off point that is analogically equivalent to the cost-effectiveness threshold.

Bearing this analogy in mind, the notion of (un)affordability can be conceptualized in several different ways. In the strict sense referred to at the beginning of this section, an intervention is unaffordable only if the cost of its adoption exceeds the entire healthcare budget; that is, if the book is thicker than the whole length of the shelf. Clearly, this situation is unlikely but not, as the case of sofosbuvir demonstrates, impossible, particularly for health systems with budgets that are relatively small at a per capita level. Alternatively, one might define an intervention as unaffordable whenever its cost exceeds the uncommitted healthcare budget; that is, if the book is thicker than the unfilled space currently vacant at the end of the shelf. This is also a very limited definition of affordability, because it ignores the possibility of removing other books to free-up the necessary space. The budget surplus typically held by most healthcare systems is extremely small or non-existent, which would make almost all new interventions, in this sense, “unaffordable.”

Neither of these understandings reflect the problem more usually faced by policymakers in deciding whether to adopt an intervention like sofosbuvir. The net cost of sofosbuvir, for most health systems, is somewhat less than the entire healthcare budget, but its budget impact is nevertheless extremely large and its associated opportunity costs are therefore very substantial. In order to make space on the shelf for a book this thick, a large number of existing books (of various heights) would need to be displaced. Strictly speaking, though such displacement may be politically and operationally challenging, there is nothing to prevent this substitution from taking place. Affordability in this sense, therefore, is a largely subjective label that indicates a health system's willingness to forego other health-generating activities in order to secure access to a new intervention. Except in extreme cases of strict unaffordability, it is determined not by budget impact, but by normative factors that influence what policymakers consider to be an acceptable type and degree of redistribution in pursuit of the goal of efficiency.

## Affordability and non-perfectionism in moral action

Once an intervention has been identified as potentially unaffordable, policymakers can (and do) respond in a variety of ways, several of which are to the disadvantage of those who would stand to benefit from the intervention in question. It has been argued that such policy responses constitute “numerical discrimination” because they lead to “patients in one group [being treated] less favorably than those in another solely because there are more in the first group than the second” (16).

The moral derivation of this argument lies in what has been called “the principle of non-perfectionism” (24). This principle posits that the mere fact that it is practically impossible for one to do all the good that one has reason to do, does not present a reason not to do whatever good one can do (24). In other words, though psychologically we might wish to achieve “completeness” in our moral action, our inability to bring about a perfect state of affairs does not constitute a reason not to bring about an improvement to the *status quo*. In the context of the affordability challenge posed by drugs like sofosbuvir, the principle of non-perfectionism suggests that the inability of the health system to offer a beneficial treatment to *all* eligible patients does not constitute a reason not to offer it to *any* eligible patients.

If one accepts the principle of non-perfectionism, then it is apparent that the fact that one intervention is ‘affordable' while another is not does not constitute a sufficient reason to discriminate between the two; what matters in this kind of situation is our duty to each individual, not their aggregation into either a small or a large patient group. Thus, all other things being equal, it is not morally permissible to prioritize between groups merely on the basis that we might meet the needs of all those in one group, as opposed to some of those in another. When faced with an “unaffordable” intervention, therefore, strategies that allow for partial provision on the same terms as any other intervention, [based, for example, on a lottery (32)], are preferable to strategies that attempt to mitigate its high budget impact by delaying or restricting its adoption, or by holding it to a more demanding standard of cost-effectiveness.

Returning for a moment to our bookshelf, the principle of non-perfectionism suggests that when faced with a sliver of empty space at the end of an otherwise full shelf, our inability to fit an entire book in the available gap does not provide a reason not to squeeze in a few individual pages. It therefore follows that when choosing between books of equal height, it would be morally impermissible to choose a thin book over a thick one purely because the thin book fits in its entirety while the thick one does not.

## A challenge: Non-marginal budget impact and the case for a modified threshold

If one accepts the principle of non-perfectionism, then it is reasonable to conclude that any approach to healthcare priority-setting that, *ceteris paribus*, uses budget impact or affordability as a criterion for choosing between interventions is potentially open to claims of numerical discrimination^3^. However, opponents of this view argue that this conclusion ignores the disproportionately large opportunity cost associated with high budget impact technologies and therefore fails to properly recognize their effect on efficiency (26).

As previously described, the cost-effectiveness threshold is a construct intended to guide decision-making by identifying the point at which an intervention's anticipated benefits are likely to exceed the health displaced by its adoption: that is, the opportunity cost. Assuming that the threshold used for decision-making accurately reflects this point, then as long as a new intervention's cost per unit health falls below this threshold, its adoption can be funded by ceasing to perform some alternative, somewhat less efficient activity. However, every time this type of substitution occurs, the marginal productivity of the health system theoretically improves by a very small amount because the least productive activity has already ceased, leading to the next intervention displacing a slightly more productive use of resources. Each subsequent new intervention therefore gives rise to a very slightly greater opportunity cost, implying a gradual lowering of the cost-effectiveness threshold.

Usually, the change to the cost-effectiveness threshold brought about by the adoption of a single new intervention will not be large enough to impact decision-making–the change to the threshold will likely be far smaller than the margin of error surrounding estimates of cost-effectiveness. However, opponents of the case against numerical discrimination argue that when an intervention with a very large budget impact is recommended, a more substantial amount of marginal activity will need to be displaced to fund it, resulting in a disproportionately large increase in opportunity cost. As a result, it is argued that such interventions shift the threshold by a material amount and must meet this more demanding threshold if they are to avoid displacing more health than they generate.

The conceptual basis for this argument has been set out in several papers (14, 17, 20, 25, 35) and also makes intuitive sense if considered through the analogy of our bookshelf. If the introduction of one very thick book displaces several books from the end of the shelf, then it will need to be taller than at least some of these books if it is to warrant its place; if it is only as tall as the shortest displaced book, then the total height of the shelved books will be diminished by its addition. It is therefore argued that assessing interventions with very large budget impacts against a more demanding cost-effectiveness threshold is justified on ethical grounds, even if this disadvantages individuals who belong to large patient groups. This is not numerical discrimination, the argument goes, because failure to modify the threshold would lead to a net reduction in population health, which, if one accepts the normative goal of efficiency, would itself be unethical.

The increased opportunity cost associated with the adoption of interventions with non-marginal budget impacts therefore appears to pose a significant challenge to the argument against numerical discrimination, at least when the potentially “discriminatory” policy involves holding large budget impact interventions to a more demanding standard of cost-effectiveness, as has sometimes been proposed (25, 26).

## A response: Breaking the link between budget impact and opportunity cost

The argument in favor of using a modified cost-effectiveness threshold for large budget impact interventions is intuitively appealing and, if one accepts the underlying assumptions, appears to be mathematically robust. However, at least one of these assumptions is potentially open to question; namely, the notion that it is both necessary and appropriate to significantly aggregate the benefits, costs and opportunity costs of an intervention's adoption when assessing its cost-effectiveness. This point can be demonstrated through a simple example.

Consider five interventions, A, B, C, D, and E, which all simultaneously undergo evaluation for potential adoption. Intervention A treats a very common condition and has a potential annual budget impact of $100 million, while interventions B, C, D, and E each treat less common conditions and have potential budget impacts of $5, 25, 30, and 40 million, respectively. The severity of the conditions treated by each of these five interventions is broadly equivalent and each has a cost-effectiveness ratio of $50,000 per quality-adjusted life-year (QALY), which is equal to the cost-effectiveness threshold in the system for which the technologies are under consideration.

Accepting the argument that intervention A's large budget impact means that it would generate greater opportunity cost than any of interventions B, C, D or E, then it would be reasonable to conclude that intervention A is not cost-effective: the cost of adopting intervention A is $50,000/QALY, compared with a cost-effectiveness threshold which will decrease to somewhat <$50,000/QALY because of the large amount of marginally cost-effective activity that must be displaced. However, the budget impact of intervention A is, of course, the same as that of interventions B, C, D and E combined, so the total opportunity cost associated with adopting these interventions, and the resulting change in the cost-effectiveness threshold, is equivalent. This reveals the flaw in the logic of accepting each of interventions B, C, D, and E as cost-effective, while rejecting intervention A as not cost-effective. The overall impact on the efficiency of the health system is the same in both scenarios–the only difference is the way in which the treated patients are aggregated into discrete groups (based, in this case, on indication and treatment).

Translated into the language of our analogy, this example illustrates the simple point that four thin books can take up the same amount of shelf space as one thick book, and that, in both cases, their insertion will lead to the same somewhat shorter books being tipped off the edge. As such, an approach to decision-making that requires that, all else being equal, the thick book be rejected while all four thin books be accepted, cannot be justified on the grounds that it maximizes book height. This example also illustrates why it might be unjust to place one thin book on the shelf in preference to part of an otherwise equivalent thick book. If we accept that books are, in principle, divisible, then prioritizing placement of a thin book over partial placement of a thick book can likewise not be justified on the grounds of efficiency. Approaches that distinguish between thick and thin books, or between “affordable” and “unaffordable” health interventions, based on arbitrary cut-offs such as “affordability caps” or “budget impact thresholds” fail to recognize this divisibility and therefore appear to constitute a form of numerical discrimination.

A more appropriate means of differentiating between interventions based on affordability could be to adopt a “sliding scale” approach, in which the cost-effectiveness threshold is gradually adjusted downwards as an intervention's budget impact increases. This avoids the need for us to act as though intervention A generates a different magnitude of opportunity cost to that of interventions B, C, D, and E combined, while also allowing for the possibility of partial adoption; if intervention A is partially adopted then the threshold would be modified downward by a smaller amount than if it were fully adopted, and by an amount equal to any other intervention with the same budget impact. However, the sliding scale approach to threshold modification nevertheless gives rise to some problems.

Consider intervention X. Intervention X has the same budget impact as intervention A and treats the same condition, but it has a slightly lower cost per QALY of $49,250. In evaluating intervention X's cost-effectiveness, an adjusted threshold of $49,000/QALY is used based on its estimated budget impact of $100 million. At this threshold, intervention X is not cost-effective. But under a sliding scale approach, the threshold for a $50 million intervention is somewhat more favorable: $49,500/QALY. As such, if treated as two equal sized subgroups, each with a budget impact of $50 million, intervention X becomes cost-effective^4^.

This example illustrates that even under a somewhat more sophisticated approach in which the cost-effectiveness threshold is adjusted downwards gradually, the apparent link between budget impact and opportunity cost is largely an artifact of how patients are aggregated. Under such an approach, the “opportunity cost” of an intervention can be reduced simply by slicing it into smaller subgroups. In some cases, it might be possible to base these subgroups on meaningful criteria, such as clinical need. However, the same effect can be seen when the criteria on which subgroups are based are entirely arbitrary. Thus, an intervention whose cost per QALY exceeds the threshold when offered to all eligible patients may become “cost-effective” if this population is split into seven smaller subgroups, based on the day of the week on which they were born.

Such an approach also ignores the wider factors that influence the marginal productivity of a health system and the many other interventions that are continuously being added and removed (28). Consider again our bookshelf. If individual pages, each paper thin and taken from a variety of different books, are continually added to (and removed from) the shelf, then the thickness of the particular book from which a page is taken ceases to bear any direct relationship with the height that each page must reach in order to warrant its place. If we accept that books can be divided into their individual pages, then while the aggregation of pages into books serves a useful practical purpose, such aggregation also potentially limits our ability to efficiently populate our bookshelf and may lead to unfairness in the allocation of resources.

But does this analogy accurately reflect reality? That is, in considering the functioning of an actual health system, do health interventions really act like collections of individual pages, or is it more accurate to treat them as discrete, largely indivisible books?

## Aggregation in theory, disaggregation in practice?

In using cost-effectiveness analysis to guide decision-making about healthcare priority-setting, the intention is to reflect the efficiency of resource use in the system as closely as possible so that choices about individual interventions can be informed by the normative goal of efficiency. Consideration therefore needs to be given to the way that benefits, costs and opportunity costs are actually incurred when an intervention is adopted. If, in reality, these impacts are realized at the whole-intervention level at a single point in time, then it may be reasonable to aggregate them in evaluating that intervention's cost-effectiveness, as is usually the case. But if the effects of an intervention's adoption are in fact phased or delayed, or are realized gradually over time, then aggregation of these effects at the point of evaluation may not accurately reflect the intervention's true impact on the health system.

Taking first the benefits associated with a new intervention's adoption, it is evident from the hypothetical examples already considered that the associated health gain is not generally delivered as a “lump sum;” rather, such gains are realized gradually, as an accumulation of the improvements in length- and quality-of-life achieved by individual patients over time. Even if we assume that the health benefits offered by an intervention are all achieved at the point of treatment, rather than in subsequent weeks, months and years (as is often the case for sofosbuvir, for example), these gains are highly disaggregated because different patients will be provided with access to the treatment at different times. Indeed, the possibility of disaggregation provides the basis for the principle of non-perfectionism, which highlights that though we may have a psychological proclivity for neat solutions, there is no sound moral reason to favor completeness in their delivery (24). In most cases it is practically feasible, and morally acceptable, to offer an intervention to some (but not all) patients, and thereby deliver some (but not all) of the possible benefits. The benefits associated with health interventions are, in reality, context-dependent and highly disaggregated.

The same is largely true for costs, which are often also context-dependent (36). Although financing arrangements for new interventions vary widely, it would be unusual for the costs of an intervention's adoption to be either paid as a single “lump sum” or realized immediately upon adoption; more likely, costs would be incurred gradually, based on the number of units purchased over time, or on pre-defined contractual milestones. If a health system has significant negotiating power, costs may even be incurred significantly after an intervention has been adopted, potentially delaying the associated opportunity cost. Costs may also vary over time, based for instance on price pressure generated by the introduction of branded competitors, or on the expiry of a patent and subsequent genericisation (37). And if a health intervention is only partially adopted, then the costs will also be partial. A decision to adopt a new intervention may, therefore, commit a health system to certain future costs, but this does not equate to all such costs being incurred immediately, at a single point in time, or steadily over a predefined period.

If the costs of a technology's adoption can be disaggregated, then it seems likely that the same would generally be true for opportunity costs. Research on the nature of the activities that are displaced to fund new interventions is limited and it is therefore difficult to speculate about how and when opportunity costs are actually incurred by different health systems (38, 39). In some cases, particularly where budget impact is extremely high, it may be that a direct causal relationship exists between the adoption of a new intervention and the decision to promptly forego one or more alternative health-generating activities. Large budget impact technologies may also be more likely than their less impactful counterparts to displace non-marginal activities: space may be made for a very thick book by removing several quite tall books, not because they are at the edge of the shelf but because they are relatively easy to identify and remove. However, in most developed health systems additional resources are almost constantly required to satisfy demand for both new and existing interventions, making it necessary–in lieu of a budget increase–for the system to continually identify low-priority activities for displacement. Opportunity costs may also be realized through less obvious types of substitution, for example by delaying access to existing treatments, deterring patients' uptake of available interventions or diluting the quality of care in an effort to free up funds for deployment elsewhere (40, 41). It may therefore be appropriate to consider the opportunity costs associated with most new interventions' adoption as taking a highly disaggregated, somewhat nebulous form, with health system administrators drawing on a constantly fluctuating pool of available (and potentially available) resources in order to support emerging higher priority activities, rather than straightforwardly replacing one specific set of activities with another every time a new intervention is adopted.

The threshold itself is also subject to several variables that may cause it to change over time. Clearly, if the budget available to the health system increases or decreases, then the cost-effectiveness threshold will move upwards or downwards based on the magnitude of this change. However, fluctuations in the threshold are also driven by several other factors including the demand for existing healthcare interventions, the efficiency of existing interventions and the development of new health technologies (13, 28).

This fluctuation in the use and availability of resources across the health system is poorly reflected in our bookshelf analogy. In reality, the adoption and displacement of health interventions within a health system does not resemble the piecemeal positioning and repositioning of discrete, well characterized books on a neatly ordered shelf. Rather, it resembles a constant flurry of largely undirected activity in which clusters of individual pages and chapters continually fly on and off an already highly disordered shelf, while policymakers with incomplete knowledge about book height, thickness and even shelf length attempt to impose order.

This is to be expected and should not be read as a critique of the bookshelf analogy itself. The analogy's purpose is to illustrate underlying principles, which it does extremely well, not to faithfully reflect the messy imperfection and complexity of the real world. However, the failure of the analogy to reflect such complexity reveals the logical flaw in any approach that really does treat healthcare interventions as inherently indivisible “books” on an otherwise unchanging shelf. Modifying the cost-effectiveness threshold based on budget impact, while ignoring myriad other variables relevant to decision-making, appears to do just that.

## Aggregation without discrimination

The starting point for this paper was the claim that approaches to healthcare priority-setting that systematically disadvantage members of large patient groups are ethically unjust: that they are a form of numerical discrimination. This claim has been challenged on the grounds that it ignores the disproportionately large opportunity costs associated with interventions that have non-marginal budget impacts. Modifying the threshold downward for interventions with very large patient populations, this argument goes, is necessary to properly reflect the opportunity cost of adopting such interventions and therefore to maintain efficiency. Such a policy is not, therefore, discriminatory.

The normative goal of efficiency is not here questioned, but this challenge is nevertheless rebutted. First, because the apparent link between budget impact and opportunity cost can be shown to be primarily an artifact of aggregation. And second, because it seems likely that the aggregation of benefits, costs and opportunity costs in theory does not reflect the way in which these effects are experienced by health systems in practice. Modification of the threshold based on budget impact therefore appears to be a blunt tool through which to estimate opportunity cost, and the systematic disadvantage at which it places members of large patient groups thus cannot be justified with recourse to the goal of efficiency. The original argument that such an approach unfairly discriminates against members of large groups appears to stand.

It could of course be argued that any form of aggregation gives rise to related challenges. No patient group is homogenous; both the costs and benefits associated with treatment vary across patient subgroups and individual patients, meaning that some may be cost-effective to treat while others are not (42, 43). And arguments concerning the disaggregation of effects are not limited to technologies with large budget impact. At the same time, some degree of aggregation is necessary if cost-effectiveness analysis is to operate as an effective tool for decision-making. So what is the appropriate level of aggregation? There is no simple answer to this question. But the logical and ethical flaw that this paper identifies in relation to budget impact highlights the need to remain cognizant of the ways in which cost-effectiveness analysis makes use of aggregation and the assumptions that underlie this practice. Such awareness ensures that we can appropriately respond to potential sources of unfairness as they arise.

## Conclusion

This paper rejects the use of threshold modification based on a technology's potential budget impact, *prima facie*, on logical and ethical grounds. However, that is not to say that attempts to more fully understand and enumerate the relationship between budget impact and opportunity cost should not be pursued. Exploring how a single intervention with a large budget impact might contribute to broader fluctuations in marginal productivity is undoubtedly a worthwhile endeavor and, as part of a deliberative process, such information has the potential to contribute important insight to decision-making. Further investigation of the ethical implications of aggregation in cost-effectiveness analysis, in theory and in practice, would also be of benefit in supporting decision-making, as would more research into how health systems respond when new interventions are adopted, and the nature of the opportunity costs incurred.

In considering such problems situated at the interface of ethics and economics, open and constructive debate of the type that motivated this paper offers a valuable opportunity to bring a range of relevant expertise to the fore in addressing them. Long may such debate continue.

## Data availability statement

The original contributions presented in the study are included in the article/supplementary material, further inquiries can be directed to the corresponding author.

## Author contributions

The author confirms being the sole contributor of this work and has approved it for publication.

## Funding

The author VC was supported in this work by a Wellcome Trust Society and Ethics Doctoral Studentship (203351/Z/16/Z).

## Conflict of interest

The author declares that the research was conducted in the absence of any commercial or financial relationships that could be construed as a potential conflict of interest.

## Publisher's note

All claims expressed in this article are solely those of the authors and do not necessarily represent those of their affiliated organizations, or those of the publisher, the editors and the reviewers. Any product that may be evaluated in this article, or claim that may be made by its manufacturer, is not guaranteed or endorsed by the publisher.

## Footnotes

^1^The question of what healthcare seeks to achieve and how well this is reflected in the logic of cost-effectiveness analysis is a matter of long debate (7–12). However, in order to illustrate its core argument as clearly and simply as possible, this paper takes the normative position that the purpose of healthcare is primarily to increase health. This should not be interpreted as a rejection of more pluralistic views and it is the opinion of the author that the argument here presented is not fundamentally undermined by these views.

^2^This excellent analogy was proposed by Tony Culyer (13) and has since been employed relatively widely as a way of describing the concepts that underlie cost-effectiveness analysis (28–31).

^3^It should be noted that the term “numerical discrimination”–as defined in this paper and in its antecedents (16, 24)–is derived from the principle of non-perfectionism and thus relates specifically to situations in which, *all else being equal*, group size and notions of “affordability” are used as criteria for prioritization. As such. this concept has relatively little to contribute to ongoing debates about the prioritization of drugs for rare and very rare diseases, in which *all else is not equal* and the role of group size is indirect. In such scenarios, small group size–so the theory goes–means a small market, which necessitates high prices, which leads to poor cost-effectiveness on a cost per QALY basis (33). As a result, patients belonging to small patient groups may routinely find themselves unable to access potentially beneficial treatments. A wide range of normative arguments have been deployed in characterizing this situation as either fair or unfair (34). However, the problem faced by policymakers in responding to the challenge of orphan drugs is ethically distinct from the “cost-effective but unaffordable” scenario considered here. In other words, failure to adopt cost-ineffective technologies for small patient groups might reasonably be considered unfair, but group size is not what drives this choice and such decisions therefore do not constitute numerical discrimination as understood by this paper.

^4^Depending on how the “sliding scale” threshold operated, it might be that only one of these subgroups would be accepted as cost-effective. If the approach to threshold modification dealt with the two subgroups sequentially, then the threshold for the second subgroup could theoretically be adjusted further downward in recognition of the interventions or services that have already been displaced by the first subgroup. But under a simplified approach–which is the more feasible option in practice–the same adjusted threshold would be applied to all interventions with a given budget impact. i.e., $100 million interventions would always be evaluated based on a threshold of $49,000/QALY, while $50 million interventions would always be evaluated based on a threshold of $49,5000/QALY.
